# New method for calculating the windward area of irregular fragments

**DOI:** 10.1038/s41598-023-48573-9

**Published:** 2024-04-24

**Authors:** Xing-yu Liu, Di-hua Ouyang, Jia-ying Wang, Zhi-yong Guo, Chun-hai Yang

**Affiliations:** 1 Engineering University of PAP, Xi’an, 710086 China; 2 Xi’an University of Architectural and Technology, Xi’an, 710055 China; 3 Changshu Institute of Technology, Changshu, 215500 China

**Keywords:** Computational science, Computer science

## Abstract

Average windward area is an important index for calculating the trajectory, velocity attenuation and terminal effect of explosive fragments. In order to solve the problems that existing theoretical method cannot calculate windward area of irregular fragment and experiment method is not convenient for automatic calculation and has low accuracy, a Monte Carlo subdivision projection simulation algorithm is proposed. The average windward area of arbitrary shaped fragments can be obtained with coordinate translation, random rotation, plane projection, convex-hull triangulation, concave boundary searching and sorting with maximum edge length constraint, subdivision area calculation, and averaging by thousands of cycles. Results show that projection area obtained by the subdivision projection algorithm is basically the same as that obtained by software method of computer aided design. Moreover, the maximum calculation error of the algorithm is less than 7%, and its accuracy is much higher than that of the equivalent ellipsoid method. The average windward area calculated by the Monte Carlo subdivision projection simulation algorithm is consistent with theoretical formula for prefabricated fragments, and the error is less than 3%. The convergence and accuracy of the Monte Carlo subdivision projection algorithm are better than those of the icosahedral uniform orientation method.

## Introduction

Average windward area is an important index for calculating the trajectory, velocity attenuation and terminal effect of explosive fragments. During explosion of energetic materials, random failure and fragmentation of packaging shell will produce unpredictable irregular shaped fragments, which makes it very difficult to calculate windward area or average windward area of irregular shaped fragments.

For the calculation of the windward areas of regularly shaped objects, Scholars have extensively studied the calculation method, such as sphere, ellipsoid, cube, cuboid, rhombus, cylinder, and hexagon. Wang^1^ and Sui^2^ established semi-empirical formulas for the windward areas of prefabricated fragments of different shapes and materials on the basis of shape coefficient, fragment mass, and correction coefficient. But this method only analyzes and summarizes windward areas of metal fragments with regular shapes, and cannot calculate non-metallic fragments or natural fragments. Quan et al.^3^ improved the calculation formula of the windward areas of sphere and cuboid fragments of tungsten and steel materials with measuring velocity. This correction method only calculates regular shaped fragments, and there is still a 7–16% error between the windward test results and the theoretical formula. Zhu et al.^4^ calculated and verified the windward areas of tri-prism fragments for different aspect ratios by establishing a rolling model. While this method can only be used to calculate the windward area of tri-prism fragments, which is not universal. Zhang et al.^5^ the calculated windward areas of cake-shaped fragments through theoretical calculation and numerical simulation, which solved the velocity attenuation of the fragments in water. This method can only solve for cake-shaped fragments and lacks generalizability. Guo et al.^6^ solved and verified the attenuation coefficient of the windward area of a V-shaped fragment with numerical simulation method. This method can only be used for prefabricated fragments whose shape is known in advance, and cannot be calculated for natural fragments. On the basis of the geometric projection method of graphics, Yang et al.^7^ established a calculation model for the windward area of spacecraft in orbit flight related to longitude, latitude, and flight attitude. However, with this method, the motion trajectory and flight attitude of the flying target need to be known in advance, and it is difficult to solve for natural fragments with unpredictable shape and flight direction.

For the calculation of the windward areas of irregularly shaped objects, no relevant theoretical formula is available. At present, there are two experiment methods to quickly calculate average windward area of irregular shaped fragments: icosahedral uniform orientation method (IUO for short) and equivalent modeling method.

The IUO method is an optical projection method which uses the projection area of sixteen directions to calculate average windward area. For example, Wang^8^, Zhang^9^, and Wei^10^ obtained projection areas of natural fragments of 16 specific directions with a test system comprising a charge coupled device (CCD for short) and subsequently the solved the approximate average windward areas of irregularly shaped fragments. However, the IUO method of optical projection suffers from three issues. First, the results of the average windward areas of the IUO method are not convergent, and the accuracy is not high. Second, the experiment method of optical projection is difficult to calculate automatically, and the workload is huge. Third, this method cannot calculate the windward areas of irregularly shaped fragments in the process of the numerical simulation of explosion and impact.

The equivalent modeling method is an approximate method of replacing irregularly shaped fragments with regular shapes with different size parameters. For example, Yang^11^ obtained the windward areas of irregularly shaped fragments with establishing an equivalent sphere model and introduced a volume-dependent coefficient $\mu$. However, a certain error exists between the equivalent windward area and the real windward area. Elvedin^12, 13^ established an equivalent ellipsoid model on the basis of the maximum and minimum diameters of natural fragments and reported that the model can quickly estimate the windward areas of arbitrarily shaped fragments. This method provides a new idea for the simulation calculation of the velocity attenuation, trajectory, and specific kinetic energy of irregular fragments, but the calculation accuracy of this method needs to be further improved. The equivalent ellipsoid model suffers from different degrees of errors with different projection angles, and the maximum error can reach 36%^12^.

In summary, the IUO method is difficult to calculate automatically and the result is not convergent; The equivalent modeling method has a large error for some fragments with large aspect ratio, and the maximum calculation error can reach 36%.

Therefore, a Monte Carlo subdivision projection simulation (MCSPS for short) algorithm is proposed in the current work, which can calculate the average windward areas of arbitrarily shaped fragments. The method involves coordinate translation, random rotation, plane projection, convex-hull triangulation, concave boundary searching and sorting with a maximum edge length constraint, subdivision area calculation, and averaging by thousands of cycles. The MCSPS algorithm have good convergence and high accuracy with a maximum error of no more than 7%. While the MCSPS algorithm also has good generality, which provides a new method for calculating dispersion characteristics parameters and terminal effect of numerical simulation fragments automatically. To verify the accuracy of the single projection area obtained by the MCSPS method, this study also compares and analyzes the results of the MCSPS algorithm and computer aided design (CAD for short) method based on recycled fragments of 48 mm and 37 mm stun grenades. The correctness of the average windward area of MCSPS algorithm is verified by comparing it with theoretical formula of regularly shaped fragments. The results of the MCSPS algorithm are also compared with equivalent modeling method by static explosion test.

## Model

**Figure 1:**
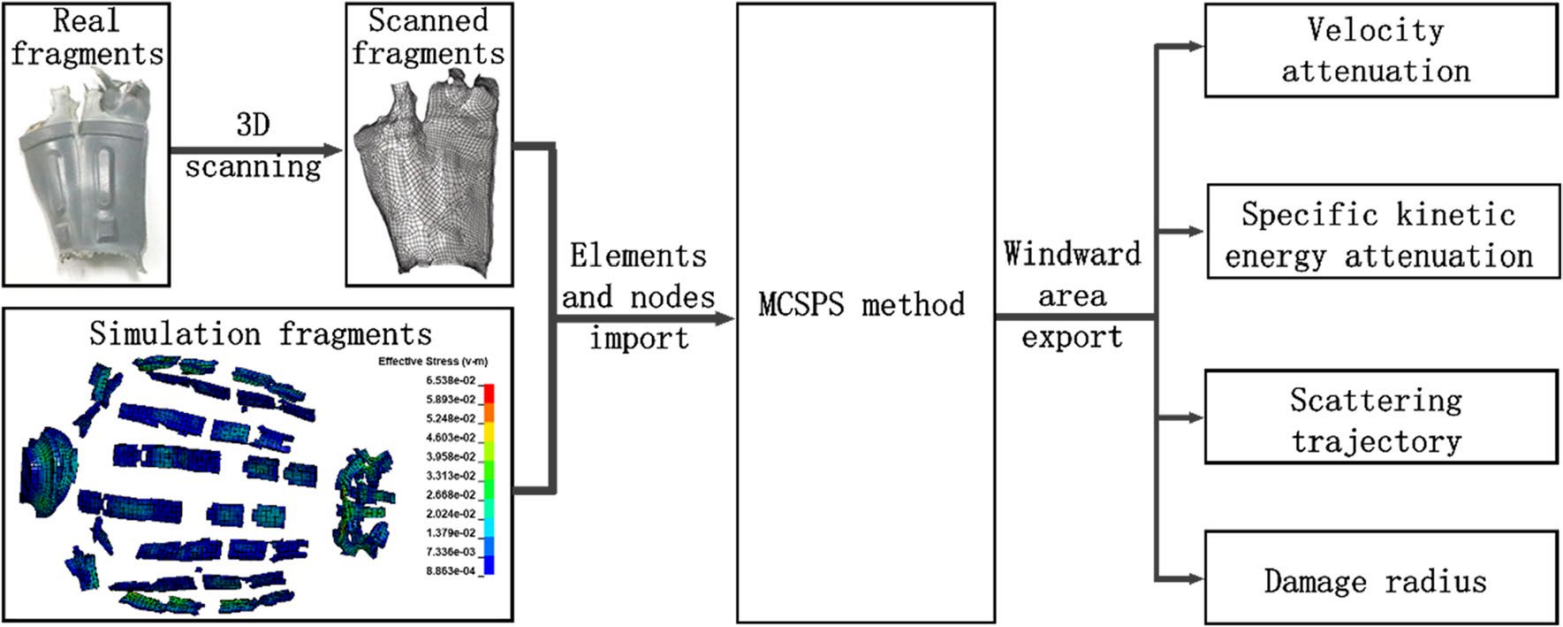
Flow chart of solving the windward areas of fragments based on the MCSPS method.

Based on the MCSPS method, the flow chart of solving the windward areas of fragments is shown in Fig. 1. On the one hand, real fragments need to be transformed into finite element fragments through three-dimensional scanning^14^. On the other hand, numerical simulation fragments can be solved directly with the MCSPS method. The coordinate matrix of elements and nodes of a whole fragment is taken as the input parameter. The average windward area of each fragment can be calculated automatically with the MCSPS method. The results can provide important parameters for the calculation of the velocity attenuation, specific kinetic energy attenuation, scattering trajectory, and damage radius of fragments.

### Model establishment

Sui^1^ and Wang^2^ established a semiempirical formula for the windward areas of prefabricated fragments with different shapes and materials by introducing shape coefficient, fragment mass, and correction coefficient. The expression is as follows:

1 $$S = k\phi m_{f}^{{2/3}}$$

where *K* is the correction factor, $\phi$ is shape coefficient, $m_{f}$ is the mass, and *S* is the average windward area.

The dimensional analysis of Eq. (1) shows that the correction factor *K* is a dimensionless unit. The dimension of windward area *S* is m^2^. The factor $m_{f}^{2/3}$ is a power function of mass, and the dimension is kg^2/3^. Thus, the fragment shape coefficient $\phi$ is a power function of density, and the dimension is (kg·m^−3^)^−2/3^. According to the dimension conversion, *S* can be regarded as a power function of volume, which is not related to the density of the material itself but is mainly associated with the geometry of the fragment. The expression is as follows:

2 $$S = f(V^{2/3} )$$

Volume is a function of spatial coordinates, that is,

3 $$V = f(x,y,z)$$

The windward area of a fragment is actually projection area of such fragment on the normal plane of a velocity vector. If the projection area of an arbitrarily shaped fragment can be calculated directly according to the spatial coordinates of the fragment nodes by using a general algorithm, then the test work of the $\phi$ value of fragments with different shapes and materials in formula (1) can be avoided so as to reduce the workload and enhance the generality. From the essence and principle of the finite element method, which is a subdivision summation method based on the iterative process of stress and strain transport in continuum mechanics, and the distance between nodes on the same fragment is not too far. Let the node coordinate matrix of the *i*-th finite element fragment be $(x_{i} ,y_{i} ,z_{i} )$. If the area of the figure surrounded by projection nodes can be obtained by some subdivision rule, then the figure area can be approximately equal to the average windward area of the finite element fragment by thousands of random projections and averaging. The expression is as follows:

4 $$\overline{A}_{i} = \mathop {\lim }\limits_{N \to \infty } \frac{{\sum\nolimits_{i = 1}^{N} {A_{{_{ij} }} } }}{N}$$

$A_{ij}$ is the graphic area of the *j*-th random projection of the *i*-th fragment, *N* is the number of random projections, and $\overline{A}_{i}$ is the average windward area of the *i*-th fragment.

According to the above analysis, the calculation model for windward areas of the MCSPS method is established. With *N* iterations of coordinate translation, random rotation, plane projection, convex-hull triangulation, concave boundary searching and sorting with a maximum edge length constraint, subdivision area calculation, and averaging by thousands of cycles, the average windward area $\overline{A}_{i}$ can be solved. When *N* approaches to infinity, the average value of the projection area will be approximately equal to the average windward area of the fragment. The expressions of each calculation step are shown in Eqs. (5) to (12).

5 $$f_{1} {:(}{\varvec{x}},{\varvec{y}},{\varvec{z}}{)}\mathop{\longrightarrow}\limits^{{{\text{Translation}}}}{(}\user2{x^{\prime}},\user2{y^{\prime}},\user2{z^{\prime}}{)}$$

6 $$f_{2} {:(}\user2{x^{\prime}},\user2{y^{\prime}},\user2{z^{\prime}}{)}\mathop{\longrightarrow}\limits^{{{\text{Rotation}}}}{(}\user2{x^{\prime}}_{{{\varvec{rot}}}} ,\user2{y^{\prime}}_{{{\varvec{rot}}}} ,\user2{z^{\prime}}_{{{\varvec{rot}}}} {)}$$

7 $$f_{3} {:(}\user2{x^{\prime}}_{{{\varvec{rot}}}} ,\user2{y^{\prime}}_{{{\varvec{rot}}}} ,\user2{z^{\prime}}_{{{\varvec{rot}}}} {)}\mathop{\longrightarrow}\limits^{{{\text{Projection}}}}{(}\user2{x^{\prime}}_{{{\varvec{proj}}}} ,\user2{y^{\prime}}_{{{\varvec{proj}}}} {)}$$

8 $$f_{4} {:(}\user2{x^{\prime}}_{{{\varvec{proj}}}} ,\user2{y^{\prime}}_{{{\varvec{proj}}}} {)}\mathop{\longrightarrow}\limits^{{{\text{Triangulation}}}}{(}{\varvec{P}},{\varvec{C}}_{convex} {)}$$

9 $$f_{5} {:(}{\varvec{C}}_{convex} {)}\mathop{\longrightarrow}\limits^{{\text{Edge - removal}}}{(}{\varvec{C}}_{concave} {)}$$

10 $$f_{6} {:(}{\varvec{P}},{\varvec{C}}_{{\text{concave}}} {)}\mathop{\longrightarrow}\limits^{{\text{Search - sort}}}{(}{\varvec{P}}_{{\varvec{b}}} {)}$$

11 $$f_{7} {:(}{\varvec{P}}_{{\varvec{b}}} {)}\mathop{\longrightarrow}\limits^{{\text{Cross - multiplication}}}{(}A_{ij} {)}$$

12 $$f_{8} {:(}A_{i1} ,A_{i2} ,...,A_{iN} {)}\mathop{\longrightarrow}\limits^{{{\text{Average}}}}{(}\overline{{A_{{\text{i}}} }} {)}$$

where $f_{1}$, $f_{2}$, and $f_{3}$ are the translation, rotation, and projection steps of the coordinate matrix, respectively; $f_{4}$ is the Delaunay convex-hull triangulation of the projection coordinate matrix, *P* is the coordinate matrix of the convex-hull triangulation nodes, and ${\varvec{C}}_{convex}$ is the node number matrix of convex-hull triangulation; $f_{5}$ and $f_{6}$ are the concave boundary searching and sorting with a maximum edge length constraint, ${\varvec{C}}_{concave}$ is the node number matrix of concave triangulation, and ${\varvec{P}}_{b}$ is the sorted boundary nodes of concave triangulation; $f_{7}$ is the area of the polygon by cross multiplication, and $A_{ij}$ is the windward area of the *i*-th fragment of the *j-*th MCSPS calculation; $f_{8}$ is the average windward area of the *i*-th fragment, and *N* is the total number of MCSPS iterations.

### Model solution

#### Coordinate transformation of fragment nodes

**Figure 2:**
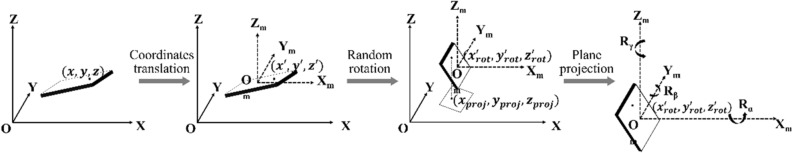
Coordinate transformation schematic of fragment nodes.

The coordinate transformation of fragment nodes involves coordinate translation, random rotation, and plane projection, as shown in Fig. 2.

##### Coordinate translation

To reduce the calculation cost of the coordinate matrix in the process of thousands of random rotations around the mass center, the origin of the rectangular coordinate system is translated into the mass center coordinate of the fragment. In this way, a local coordinate system is established to improve calculation efficiency. The expression is as follows:

13 $${(}\user2{x^{\prime}},\user2{y^{\prime}},\user2{z^{\prime}}{)}^{{\text{T}}} { = (}{\varvec{x}},{\varvec{y}},{\varvec{z}}{)}^{{\text{T}}} - (x_{m} ,y_{m} ,z_{m} )^{{\text{T}}}$$

where ${(}{\varvec{x}},{\varvec{y}},{\varvec{z}}{)}$ is the nodes coordinate matrix of the fragment, $(x_{m} ,y_{m} ,z_{m} )$ is the center of the mass coordinate of the fragment, ${(}\user2{x^{\prime}},\user2{y^{\prime}},\user2{z^{\prime}}{)}$ is the node coordinate matrix after translation, and $(x^{\prime},y^{\prime},z^{\prime})$ is any node coordinate in the fragment after translation.

##### Coordinate rotation

Assume that any flight attitude of each fragment in the dispersion process is an equal probability event. Then, a group of angular variables of random rotation $(\alpha_{ij} ,\beta_{ij} ,\gamma_{ij} )$ can be generated by the pseudo-random number method. These variables are subject to a uniform distribution in the interval $[0,2\pi ]$ and rotate around three coordinate axes (*X*, *Y*, and *Z*). With matrix transformation rule, the vector expression of fragment node rotating around three local coordinate axes in turn is obtained as follows:

14 $$\left[ {\begin{array}{*{20}c} {x^{\prime}_{rot} } \\ {y^{\prime}_{rot} } \\ {z^{\prime}_{rot} } \\ \end{array} } \right] = \left[ {\begin{array}{*{20}c} {cos\gamma_{ij} } & {sin\gamma_{ij} } & 0 \\ { - sin\gamma_{ij} } & {cos\gamma_{ij} } & 0 \\ 0 & 0 & 1 \\ \end{array} } \right] \cdot \left[ {\begin{array}{*{20}c} {cos\beta_{ij} } & 0 & { - sin\beta_{ij} } \\ 0 & 1 & 0 \\ {sin\beta_{ij} } & 0 & {cos\beta_{ij} } \\ \end{array} } \right] \cdot \left[ {\begin{array}{*{20}c} 1 & 0 & 0 \\ 0 & {cos\alpha_{ij} } & {sin\alpha_{ij} } \\ 0 & { - sin\alpha_{ij} } & {cos\alpha_{ij} } \\ \end{array} } \right] \cdot \left[ {\begin{array}{*{20}c} {x^{\prime}} \\ {y^{\prime}} \\ {z^{\prime}} \\ \end{array} } \right]$$

where $(x^{\prime},y^{\prime},z^{\prime})$ is the coordinate vector of any node of a fragment after translation, $(\alpha_{ij} ,\beta_{ij} ,\gamma_{ij} )$ are the random variables of the *j*-th rotation of the *i*-th fragment, and $(x^{\prime}_{rot} ,y^{\prime}_{rot} ,z^{\prime}_{rot} )$ is the coordinate vector of any node of a fragment after rotation.

##### Coordinate projection

Let any node coordinate of a fragment after random rotation be $(x^{\prime}_{rot} ,y^{\prime}_{rot} ,z^{\prime}_{rot} )$, and let the node coordinate after projection to a space plane be $\left( {x_{proj} ,y_{proj} ,z_{proj} } \right)$. According to plane point-normal equation and the parallel principle of normal and projection vectors, the following expression can be obtained:

15 $$\left\{ \begin{gathered} x_{s} \left( {x^{\prime}_{proj} - x_{0} } \right) + y_{s} \left( {y^{\prime}_{proj} - y_{0} } \right) + z_{s} \left( {z^{\prime}_{proj} - z_{0} } \right) = 0 \hfill \\ y^{\prime}_{proj} = \frac{{y_{s} }}{{x_{s} }}\left( {x^{\prime}_{proj} - x^{\prime}_{rot} } \right){ + }y^{\prime}_{rot} \hfill \\ z^{\prime}_{proj} = \frac{{z_{s} }}{{x_{s} }}\left( {x^{\prime}_{proj} - x^{\prime}_{rot} } \right){ + }z^{\prime}_{rot} \hfill \\ \end{gathered} \right.$$

where $(x^{\prime}_{rot} ,y^{\prime}_{rot} ,z^{\prime}_{rot} )$ is any node coordinate of a fragment after random rotation, $\left( {x^{\prime}_{proj} ,y^{\prime}_{proj} ,z^{\prime}_{proj} } \right)$ is the node coordinate after projection, $\left( {x_{0} ,y_{0} ,z_{0} } \right)$ is a point coordinate on the projection plane, and $\left( {x_{s} ,y_{s} ,z_{s} } \right)$ is the normal vector of the projection plane.

#### Delaunay convex-hull triangulation

**Figure 3:**
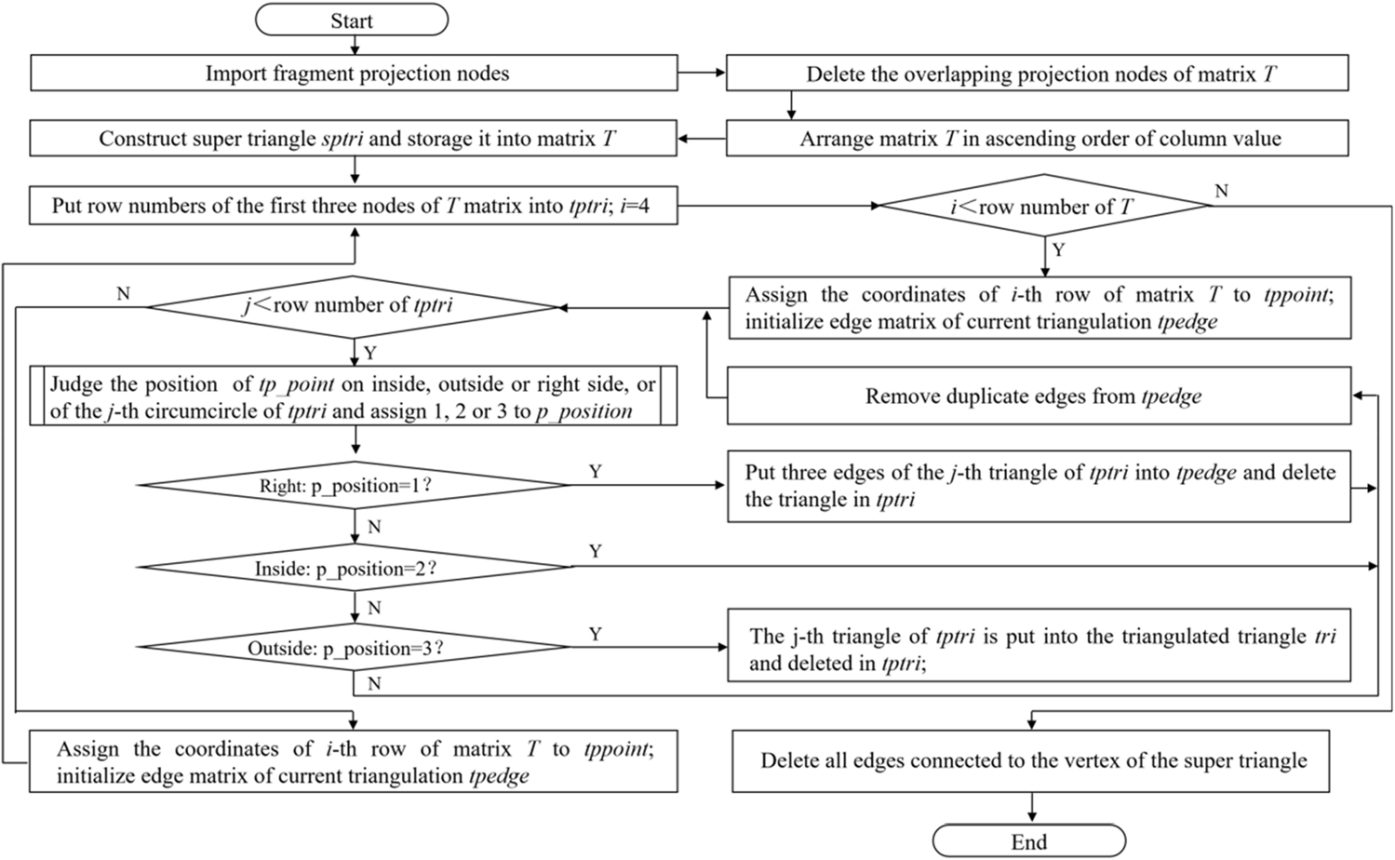
Flow chart of Delaunay convex-hull triangulation.

The Delaunay triangulation of convex-hull nodes mainly involves incremental insertion, divide-conquer, and triangulation growth^15^. The current work adopts an improved Bowyer–Watson incremental insertion algorithm^16^, which is more efficient than the Lawson algorithm. The improved algorithm shows better robustness than the standard Bowyer–Watson algorithm. The improved Bowyer–Watson algorithm can prevent the center of the circumscribed circle from falling on the outside of the standard super triangle of a large obtuse angle by introducing a magnification factor to ensure that the subdivision boundary is a complete convex-hull. By judging the position of the insertion point on the inside, outside, or right side of the subdivided triangle, the current triangulation is put on the stack, re-subdivided, or added to the next triangulation correspondingly. Finally, the Delaunay triangulation mesh can be established by the loop that satisfies the characteristics of an empty circumcircle and minimum angle maximization. The algorithm flow is shown in Fig. 3.

In the evaluation of the position of a point coordinate $(x_{i} ,y_{i} )$, the circumscribed circle center coordinate $(x_{{\text{o}}} ,y_{o} )$ and radius *r* need to be solved with three vertex coordinates $(x_{{i{ - }1}} ,y_{{i{ - }1}} )$, $(x_{{i{ - }2}} ,y_{{i{ - }2}} )$, and $(x_{{i{ - 3}}} ,y_{{i{ - 3}}} )$ of the triangle. With an equal distance between three points on the circle and its center, the calculation expressions of the center can be derived as formulas (16) and (17). With the distance relationship between the insertion point and the circle center, the symbol function expression of the insertion point position can be obtained as formula (18)^17^. When *p_positon* = 1, the insertion point $(x_{i} ,y_{i} )$ is on inside of the triangle; when *p_positon* = 2, the insertion point $(x_{i} ,y_{i} )$ is on outside of the triangle; when *p_positon* = 3, the insertion point is on the right side of the triangle.

16 $$x_{o} = \frac{{(y_{i - 2} - y_{i - 3} )(y_{i - 1}^{2} - y_{i - 3}^{2} + x_{i - 1}^{2} - x_{i - 3}^{2} ) - (y_{i - 1} - y_{i - 3} )(y_{i - 2}^{2} - y_{i - 3}^{2} + x_{i - 2}^{2} - x_{i - 3}^{2} )}}{{2 \cdot [(x_{i - 1} - x_{i - 3} )(y_{i - 2} - y_{i - 3} ) - (x_{i - 2} - x_{i - 3} )(y_{i - 1} - y_{i - 3} )]}}$$

17 $$y_{o} = \frac{{(x_{i - 2} - y_{i - 3} )(x_{i - 1}^{2} - x_{i - 3}^{2} + y_{i - 1}^{2} - y_{i - 3}^{2} ) - (x_{i - 1} - x_{i - 3} )(x_{i - 2}^{2} - x_{i - 3}^{2} + y_{i - 2}^{2} - y_{i - 3}^{2} )}}{{2 \cdot [(y_{i - 1} - y_{i - 3} )(x_{2} - x_{i - 3} ) - (y_{i - 2} - y_{i - 3} )(x_{i - 1} - x_{i - 3} )]}}$$

18 $$p\_position = \left\{ {\begin{array}{*{20}l} {{3, }\qquad x_{i} - x_{o} > r} \hfill \\ {{1},\qquad (x_{i} - x_{o} )^{2} + (y_{i} - y_{o} )^{2} < r^{2} } \hfill \\ {{2},\qquad {\text{others}}} \hfill \\ \end{array} } \right.$$

#### Concave boundary searching and sorting with a maximum edge length constraint

**Figure 4:**
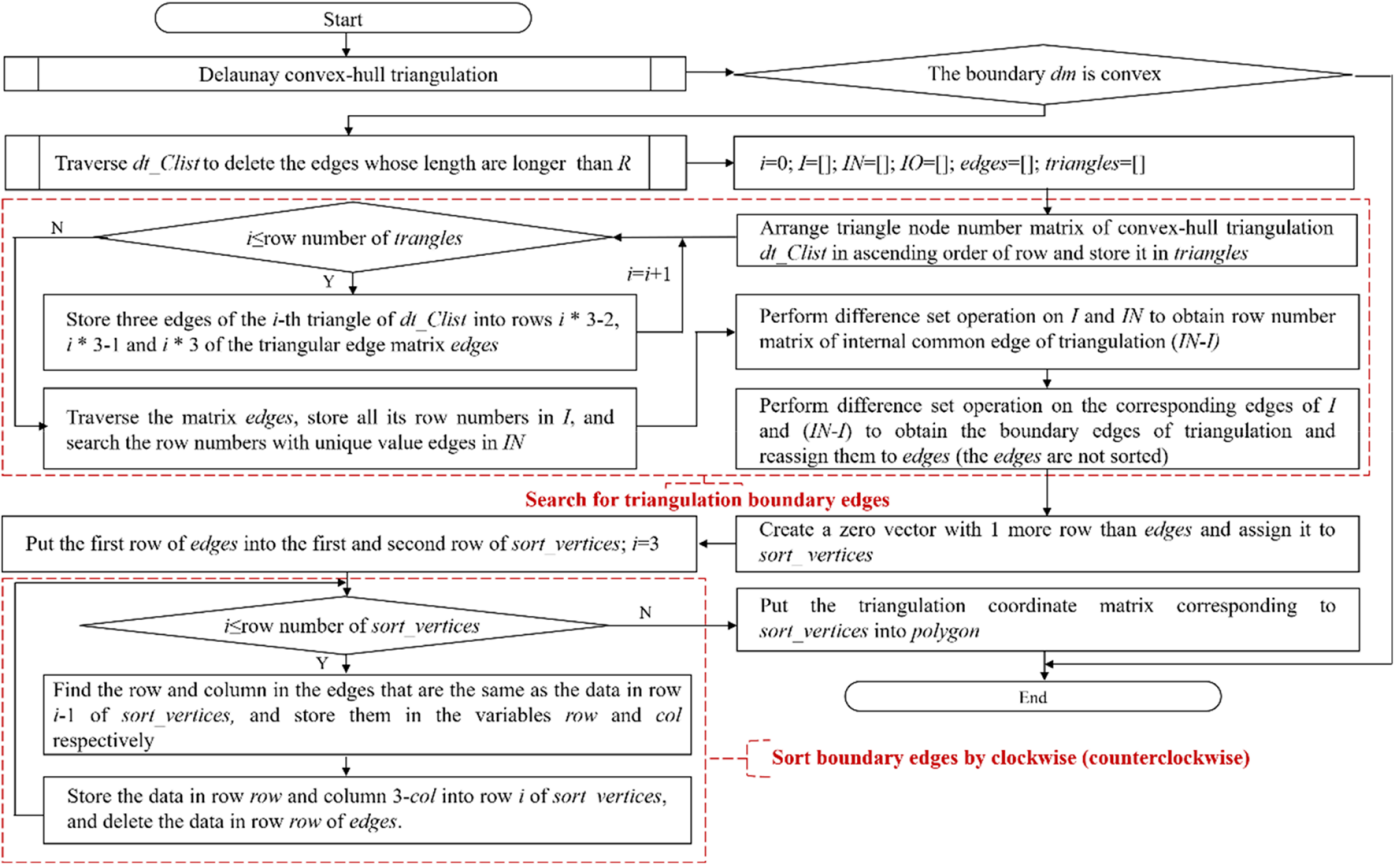
Flow chart of concave boundary searching and sorting with a maximum edge length constraint.

The Delaunay triangulation of the improved Bowyer–Watson algorithm is convex-hull triangulation, and its boundary area is larger than that of a fragment with a concave feature. By analyzing the triangular mesh characteristics of the Bowyer–Watson algorithm, this work finds that although the distance between the boundary nodes of some concave regions is considerably large, the triangular mesh is still forcibly constructed with the convex-hull subdivision principle. Hence, the average length of the inner edges is far less than that of the boundary edges. According to this feature, concave boundary searching and sorting with a maximum edge length constraint is proposed herein. This step involves distinguishing the concavity and convexity of the subdivision boundary, removing the subdivision edge exceeding the constraint length, searching the subdivision boundary, and sorting the boundary. The algorithm flow chart is shown in Fig. 4.

As shown in Fig. 4, the Delaunay convex-hull triangulation can transform the discrete projection nodes *T* into a Delaunay triangulation according to the rule of an empty circumscribed circle, and the convex-hull boundary *dm* and triangle matrix *dt_Clist* can be obtained. The concavity or convexity of the division boundary are judged with the principle of whether an edge larger than *R* exists in the convex-hull triangulation. This principle can improve the calculation efficiency when the fragment projection is a convex-hull. *R* is defined as *λ*_scale_ times of the average edge length as a constraint to remove the long edges and obtain the concave boundary. Concave boundary searching can obtain boundary edges on the basis of a difference set operation between the common edges and all edges. Boundary edge sorting can obtain the sorted polygon vertices according to the search traversal of stacking and popping. The cross-multiplication method is used to solve the arbitrary polygon area.

*T* is the projection node set without overlapping nodes, *dm* is the boundary of the convex-hull triangulation, *dt_Clist* is the node number of the convex-hull triangulation triangle, *edges* is the boundary of the convex-hull triangulation in ascending order, *I* is the row numbers set of *edges* without repeated edges, *IN* is the row numbers set of *edges*, *IO* is the row numbers set of the boundary edges, *sort_vertices* is used to store the sequence of boundary vertices, and *polygon* denotes the coordinates matrix of the boundary edge nodes sorted clockwise.

The value of *R* is defined as follows:

19 $$R{ = }\frac{{\lambda_{{{\text{scale}}}} \sum\limits_{i = 1}^{{n_{e} }} {l_{i} } }}{{n_{e} }}$$

where *R* is edge length constraint, $\lambda_{{{\text{scale}}}}$ is magnification factor, $l_{i}$ is the length of *i*-th edge of the triangulation, and *n*_*e*_ is the total number of triangulation edges. According to the rule of the circumscribed circle of the Delaunay convex-hull triangulation and the distance characteristics of finite element of the regular hexahedral, the triangular edges with the non-concave nodes can be removed well when $\lambda_{{{\text{scale}}}}$ is set to 4–6. In this work, $\lambda_{{{\text{scale}}}}$ is set to 4.

The difference set calculation expression for the boundary search of concave triangulation is as follows:

20 $$E_{IO} = E_{I} - E_{IN - I}$$

where ***E***_***IO***_ is the boundary edge set of concave polygon area with only one common edge, ***E***_***IN-I***_ is the inner edge set of concave polygon region with two common edges, and ***E***_***I***_ is a collection of concave polygon edges without duplicate edges.

For some irregular objects with prominent concave features, a cavity-like mesh area is probably formed under a certain angle projection. The average edge length of the concave mesh is closer to the length of the cavity-like invalid edge. When the invalid edges are deleted by edge length constraint *R*, effective edges inside the concave mesh are deleted at the same time, resulting in the formation of a new cavity area or non-closed edge. To obtain the cavity-like mesh boundary, the set ***E***_***IO***_ containing cavity edge, unclosed edge and boundary edge is assigned to the stack variable ***lines*** and searched based on the principle that "the number of boundary edge nodes must be closed from beginning to end". The search strategy is as follows:

1. Search from the first edge in the ***lines***. When no edge is connected to the end of the *i*-th edge, it is judged as a non-closed edge, and the *i*-th edge is removed from the boundary edge sequence.
2. When one or more edges are connected to the *i*-th edge, the first connected edge is removed from the stack ***E***_**IO**_ and added to the stack ***lines***.
3. If the number of nodes in the obtained closed sequence is too little (less than or equal to half of ***E***_***IO***_), the closed sequence is judged to be a cavity and thus needs to be discarded. The search process is then initiated from the ***lines*** again.

With the above search strategy, the coordinate matrix of boundary nodes ***polygon*** can be obtained. The subdivision area *A*_*ij*_ can be solved by cross multiplication, which is a calculation method of polygon area. The formula is as follows^18^:

21 $$A_{ij} = \frac{1}{2} \cdot \left| {\sum\limits_{h = 1}^{n - 1} {{(}x_{h} {,}y_{h} {)} \times {(}x_{h + 1} {,}y_{h + 1} {)}} { + (}x_{n} {,}y_{n} {)} \times {(}x_{1} {,}y_{1} {)}} \right|$$

where $A_{ij}$ is the polygon area, ${(}x_{h} {,}y_{h} {)}$ is the coordinate of *h*-th node, ${(}x_{h + 1} {,}y_{h + 1} {)}$ is the coordinate of *h* + 1 node, and *n* is the number of the polygon vertices.

## Verification and comparison

### Area verification of subdivision projection algorithm

**Figure 5:**
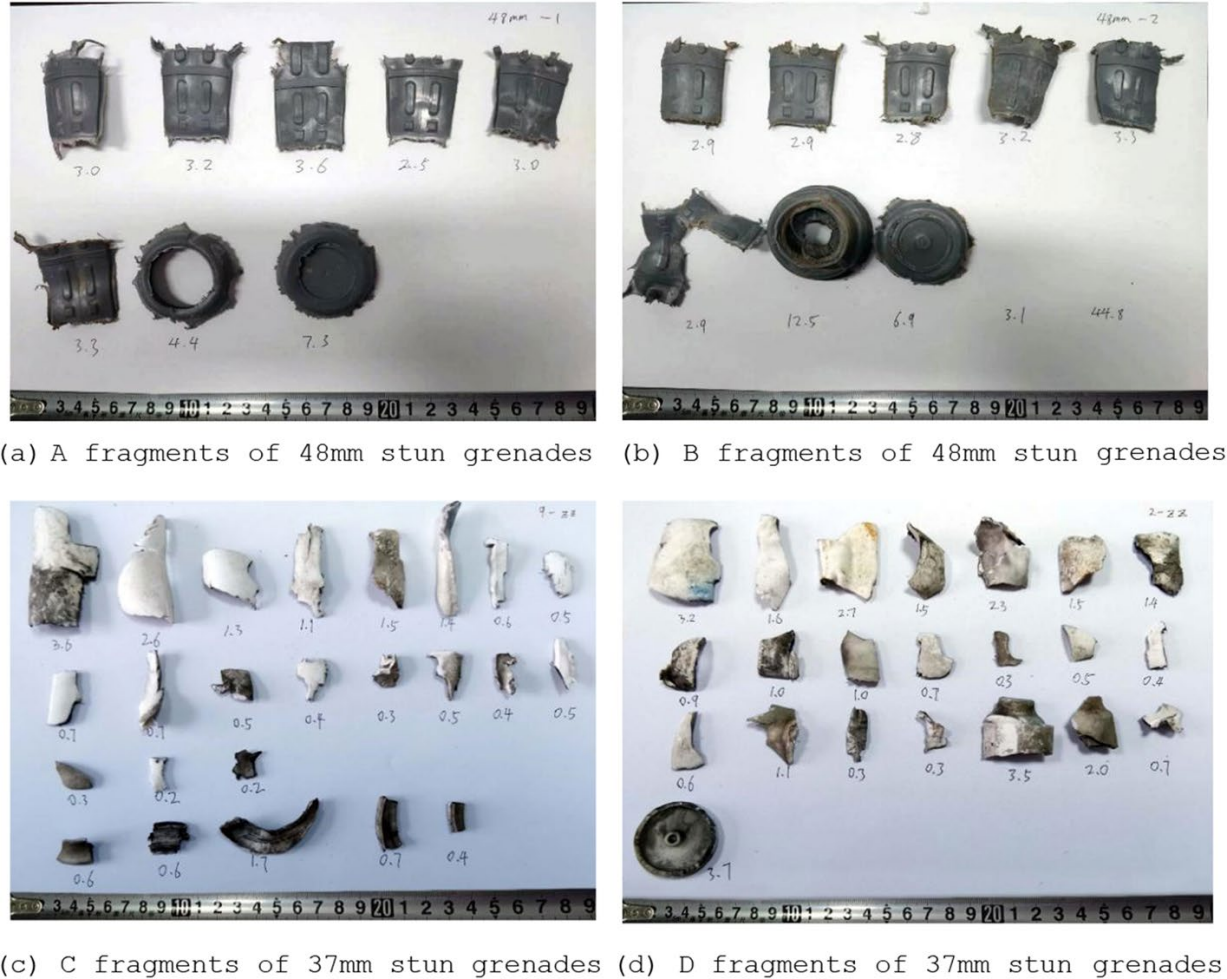
Irregular fragment samples.

**Figure 6:**
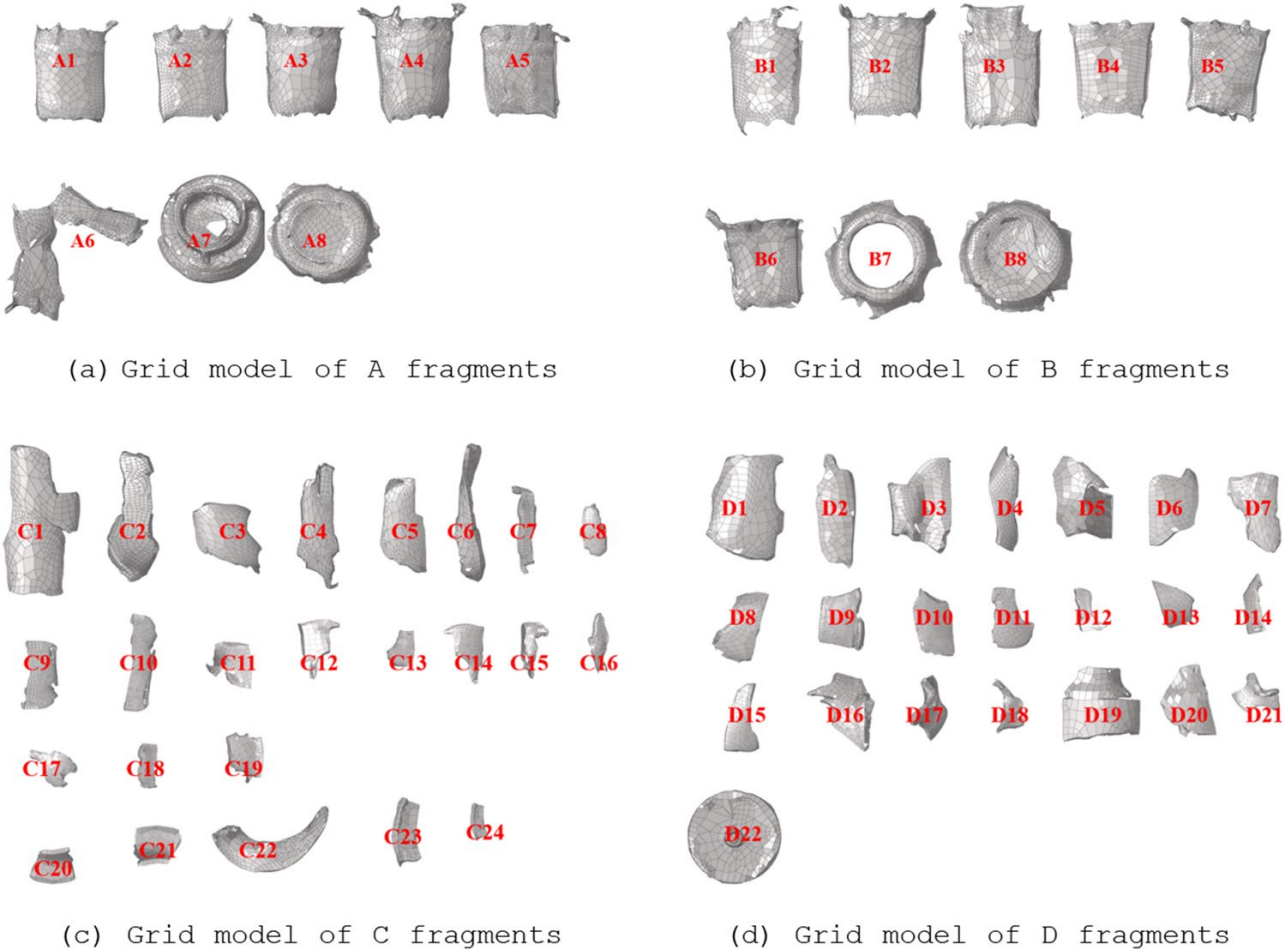
Irregular fragment grid model.

To verify the calculation accuracy of subdivision projection algorithm, the projection areas under different rotation angles are compared by CAD software method and subdivision projection algorithm. The irregularly shaped fragments of four 48 mm and 37 mm stun grenades are selected as the samples, which were recorded as A, B, C, D. The real fragments and equal scale grid model are shown in Figs. 5 and 6.

**Figure 7:**
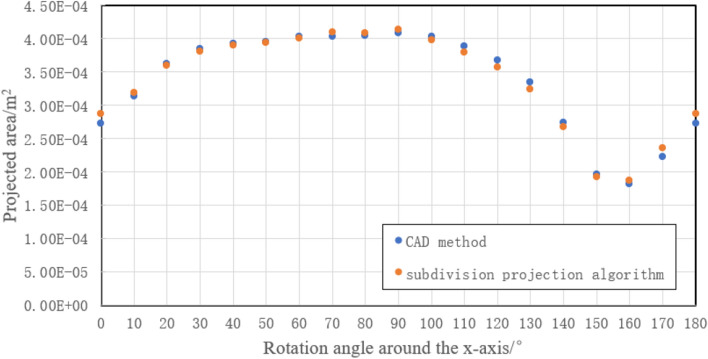
Comparison of projection areas obtained by the CAD method and subdivision projection algorithm when A1 is rotated around the x-axis.

**Figure 8:**
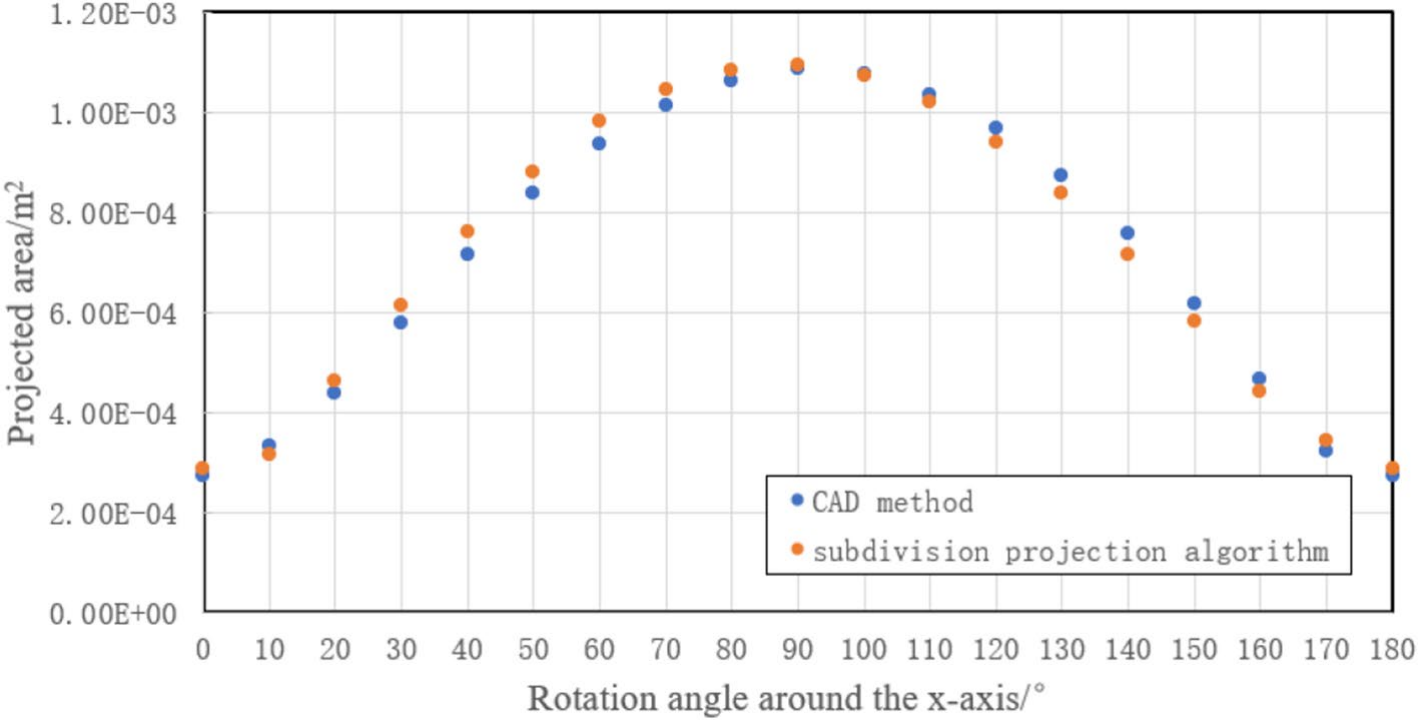
Comparison of projection areas obtained by the CAD method and subdivision projection algorithm when A1 is rotated around the y-axis.

Considering the projection symmetry, the fragments are respectively rotated around the x-axes and y-axes in 10° increments in the range of 0°–180° and then projected to the X-O-Y plane. To facilitate the presentation of process data with MCSPS method, A1 fragment was analyzed as an example. The comparison result of the projection areas obtained by the CAD method and subdivision projection algorithm for A1 are shown in Figs. 7 and 8. For A1, the projection area obtained by subdivision projection algorithm is close to that obtained by the CAD method, with the error being less than 7%. The projection area obtained by the equivalent ellipsoid method is affected by fragment shape, resulting in a maximum calculation error of 36% at a certain projection angle^12^. The results show that the calculation accuracy of the MCSPS algorithm is higher than the equivalent ellipsoid method.

Similarly, the projection areas and errors of other fragments are calculated under different angles. The maximum errors of A, B, C, D fragments rotating around the x-axes and y-axes are obtained, and some of the data is shown in the figure in Table 1. The results of C and D are very close to the results of the CAD software method, and the error is less than 2%. The maximum calculation errors of A and B are larger than those of C and D, but the maximum error does not exceed 7%. This result shows that the calculation accuracy of the fragments with planar feature is higher than that with prominent surface feature for the subdivision projection algorithm.

**Table 1:** Comparison of maximum errors of sample fragments in the range of 0°–180°

Fragment	Number of nodes	Maximum error of rotation around the x-axis	Maximum error of rotation around the y-axis (%)
A1	8061	5.89	6.52
A2	9011	5.23	5.51
A3	7232	4.55	5.32
A4	8520	5.93	6.21
A5	9157	6.13	5.97
B1	6763	6.82	4.77
B2	8852	5.09	6.23
B3	8920	5.13	5.53
B4	6985	4.22	5.42
B5	8752	5.68	5.37
C1	5869	0.54	0.87
C2	7032	0.79	0.56
C3	3326	0.25	0.57
C4	4258	1.45	1.26
C5	4760	0.97	1.02
D1	5473	0.35	0.35
D2	3803	0.82	0.88
D3	4025	1.02	1.21
D4	2890	0.66	0.52
D5	3158	0.93	0.91

**Figure 9:**
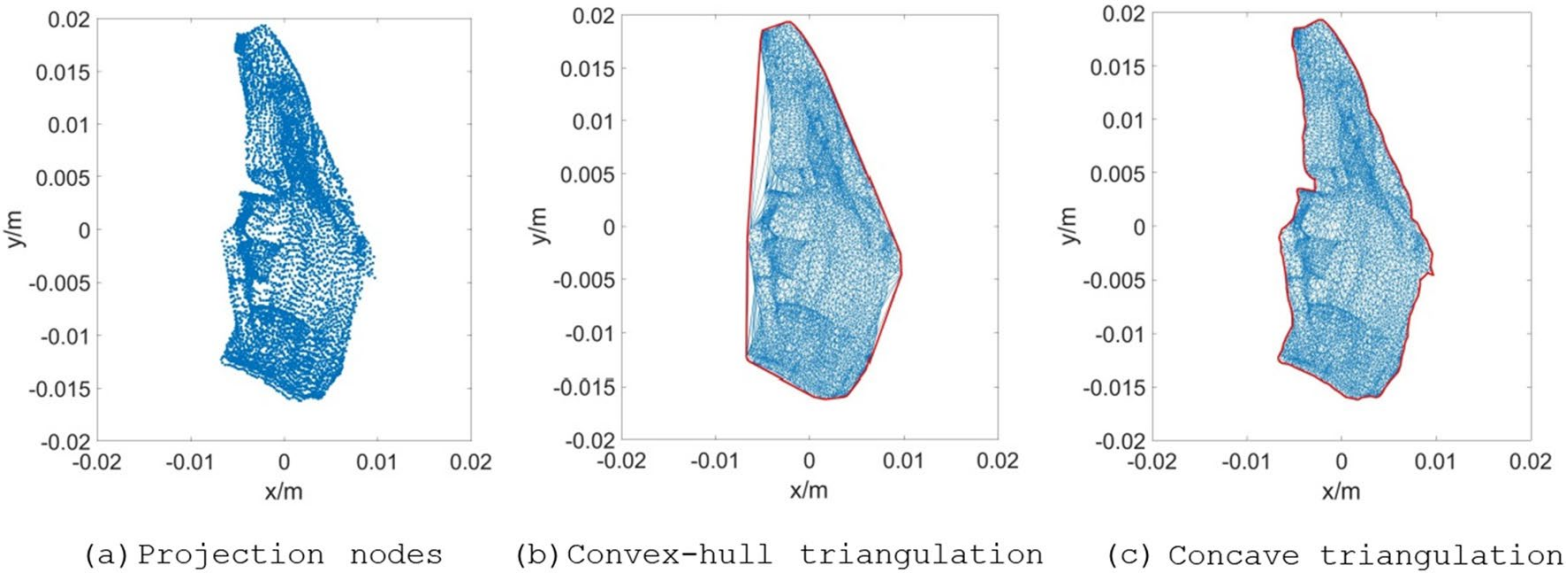
A1 subdivision projection graphics rotated 170° around the x-axis.

**Figure 10:**
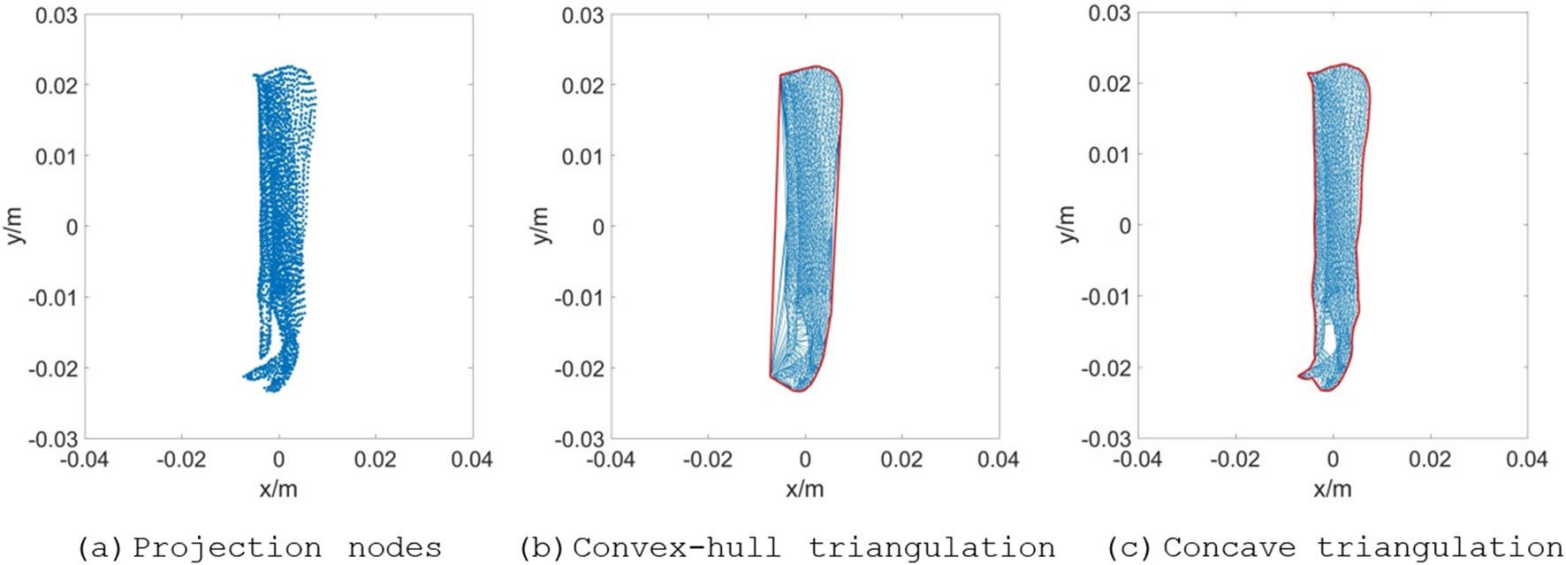
A1 subdivision projection graphics rotated 50° around the y-axis.

In analyzing the reasons for the error, two main calculation errors are considered. First, the outer angle of some polygon concave boundaries is a small acute angle, as shown in Fig. 9. The average length of triangulation edge at this acute angle is close to the average length of the whole triangulation, and the search algorithm cannot completely remove it. But the error can be further reduced by appropriately reducing the constraint value *R*. Second, a cavity or cavity-like figure exists in triangulation area, as shown in Fig. 10. The triangulation edges at the cavity-like closure zone are smaller than average length of the whole triangulation, which cannot be automatically removed. In this case, the edges are also used as the boundary edge when searching for the boundary edge, thereby resulting in an enlarged boundary area and the formation of calculation errors. Among them, C and D fragments are more planar in shape, and there is no small acute angle or cavity-like area during projection. The subdivision projection algorithm can better search the real boundary of fragment nodes. Therefore, compared with C and D, the error between the obtained results and the software results are smaller.

### Verification of average windward area of MCSPS algorithm

**Figure 11:**
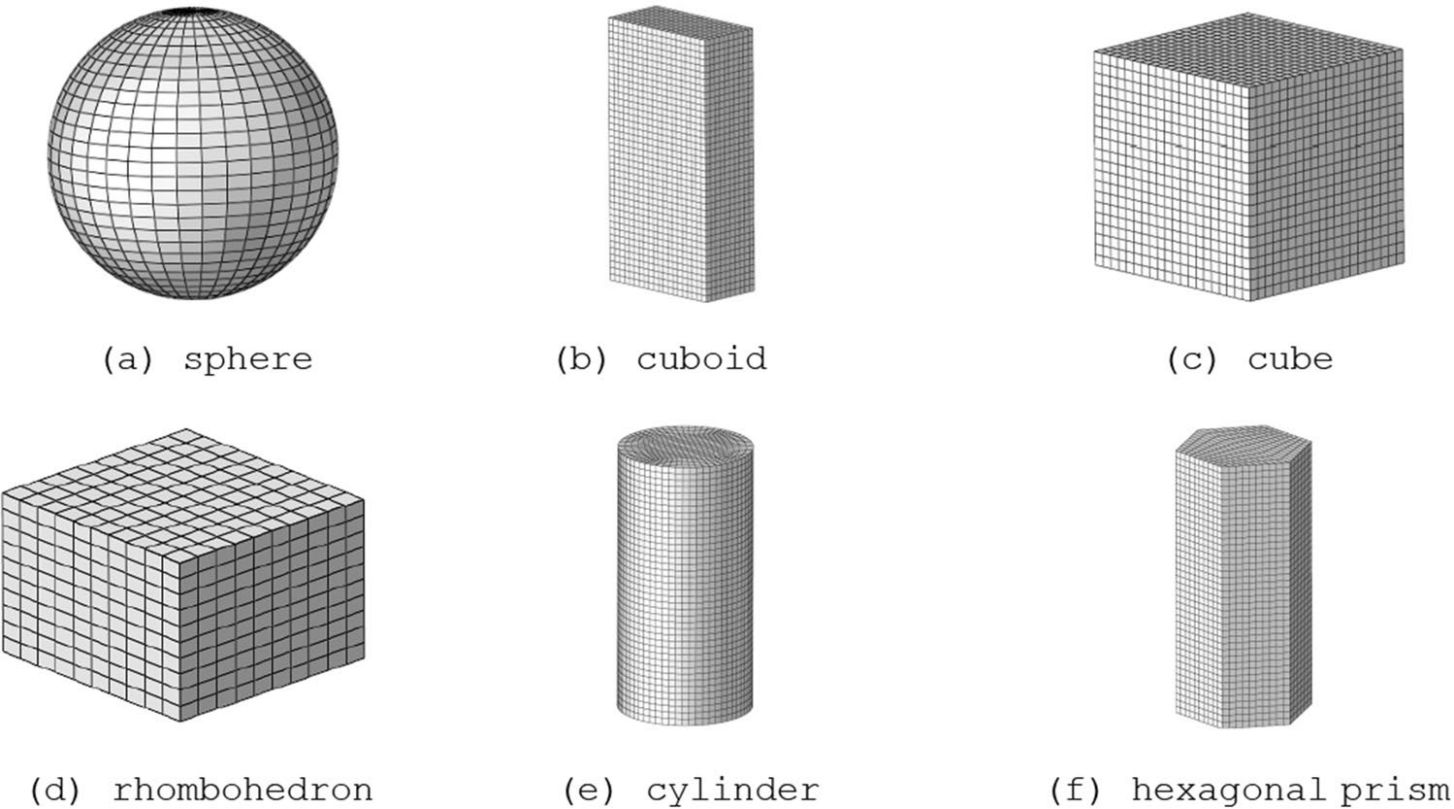
The mesh models of six regularly shaped fragment.

To verify the correctness of the average windward area obtained by the MCSPS algorithm after thousands of random projections, six prefabricated fragments of regular shape such as sphere, cuboid, cube, rhombohedron, cylinder and hexagonal prism are used as samples. The results of the MCSPS algorithm are compared with the theoretical formula of the regularly shaped fragments. The mesh models of the regularly shaped fragments are shown in Fig. 11.

The geometric characteristics and area of regularly shaped fragments are analyzed, respectively. The projection area of the spherical fragment is always its maximum cross-sectional circle area. The projection areas of the cube, cuboid, and rhombohedron fragments in a certain spatial attitude are actually the sums of the two directions cosine of the areas of the three rectangular or rhombic surfaces in the lower half. The projection area of the cylindrical fragment is the sums of the cosine of the areas of a complete circle surface of the cylinder and the largest longitudinal section. The projection area of the regular hexagonal prism fragment is the sum of the cosine of the areas of the complete regular hexagon and the largest longitudinal section. By integrating the directional cosine from 0° to 90° and taking the average, the theoretical formula for the average projection area of each fragment can be derived. On the basis of the above analysis, the formula for calculating the average windward areas of the prefabricated fragments of sphere, cuboid, cube, rhombohedron, cylinder, and hexagonal prism are as follows^8^:

22 $${\text{cuboid fragment}}\;\;\overline{S} = \frac{4}{{\pi^{2} }}ab{ + }\frac{4}{{\pi^{2} }}bc{ + }\frac{4}{{\pi^{2} }}ca$$

23 $${\text{cube fragment}}\;\;\overline{S} = \frac{12}{{\pi^{2} }}a^{2}$$

24 $${\text{rhombohedron}}\;{\text{fragment}}\;\;\overline{S} = \frac{4}{{\pi^{2} }}a^{2} \sin \theta { + }\frac{8}{{\pi^{2} }}ab$$

25 $${\text{cylindrical fragment}}\;\;\overline{S} = \frac{2}{\pi }\pi r^{2} { + }\frac{2}{\pi }2rh = 2r^{2} + \frac{4}{\pi }rh$$

26 $${\text{hexagonal prism fragment}}\;\;\overline{S} = \frac{2}{\pi } \cdot \frac{3\sqrt 3 }{2} \cdot a^{2} { + }\frac{2}{\pi } \cdot 2a \cdot h = \frac{3\sqrt 3 }{\pi }a^{2} + \frac{4}{\pi }ah$$

where *a*, *b*, and *c* are the edge length of the fragments, *θ* is the included angle of the diamond surface (acute angle), *h* is the height of the cylinder and regular hexagonal prism, *r* is the radius of the cylinder fragment.

The nodes data of six regularly shaped fragment are substituted into the MCSPS algorithm for calculation, and the results are compared with the theoretical values of Eqs. (22) to (26). The cycle number of the MCSPS algorithm is set to 16,384 (2^14^), and the average mesh size of each regularly shaped fragment is 1 mm × 1 mm × 1 mm. The comparison results of the average windward areas are shown in Table 2, which obtained by the MCSPS algorithm and the theoretical values. The results of the MCSPS algorithm for the six regularly shaped fragments are in good agreement with the theoretical values, and the maximum calculation error is ≤ 3%. Hence, the average windward area obtained by the MCSPS algorithm is accurate and reliable.

**Table 2:** Comparison of average windward area between MCSPS algorithm and theoretical formula.

Fragment type	Feature size	Mesh size	Number of nodes	MCSPS/m^2^	Theoretical value/m^2^	Error (%)
Sphere	*r* = 1 cm	1 mm × 1 mm × 1 mm	6385	3.105E−04	3.142E−04	1.18
Cuboid	*a* = 1 cm, *b* = 2 cm, *c* = 4 cm	1 mm × 1 mm × 1 mm	9471	5.667E−04	5.674E−04	0.12
Cube	*a* = 2 cm	1 mm × 1 mm × 1 mm	9261	4.861E−04	4.863E−04	0.04
Rhombohedron	*a* = 1 cm, *b* = 1 cm, θ = π/3	1 mm × 1 mm × 1 mm	1331	1.157E−04	1.162E−04	0.43
Cylinder	*r* = 1 cm, *h* = 4 cm	1 mm × 1 mm × 1 mm	21,320	6.917E−04	7.093E−04	2.48
Hexagonal prism	*a* = 1 cm, *h* = 4 cm	1 mm × 1 mm × 1 mm	9471	6.584E−04	6.747E−04	0.55

There are two main reasons for the results error of MCSPS algorithm and theoretical formula. The first is the model error. As the mesh model is the subdivision and reconstruction of real objects, the mesh size directly affects the accuracy of the geometric features of objects, especially for geometric objects such as sphere and cylinder with curves and surfaces. The large mesh size causes the cylinder to be described as a regular polygon prism and the sphere to be described as a regular polyhedron. Therefore, the calculation errors of the cylinder and sphere fragments with curved surface characteristics are greater than that of the other fragments. For example, the error of the cylinder in Table 1 reaches 2.48%. The second is the pseudo-random error. The random number of uniform distributions generated on the basis of the software method is a pseudo-random number. An error inevitably exists between the simulated random rotation angle sample and the actual uniform distribution, but the error can gradually decrease with the increase of the cycle number, such as the calculation errors of the cuboid, cube, rhombohedron, and regular hexagonal prism fragments.

### Comparison of MCSPS algorithm and IUO method

The IUO is a common method for calculating the average windward area of irregular fragments. The method usually averages the projection areas in 16 specific directions of an icosahedron on the basis of a CCD optical system. However, the convergence, calculation accuracy, and solution time of this method need to be further compared and verified. Therefore, D1 is taken as a sample in this section to compare the convergence, calculation accuracy and solution time of MCSPS algorithm and IUO method. The operating environment is Intel(R) Core(TM) i7-8750 h CPU @ 2.20 GHz, 16 GB, and MATLAB r2017a.

#### Comparison of convergence and calculation accuracy

**Figure 12:**
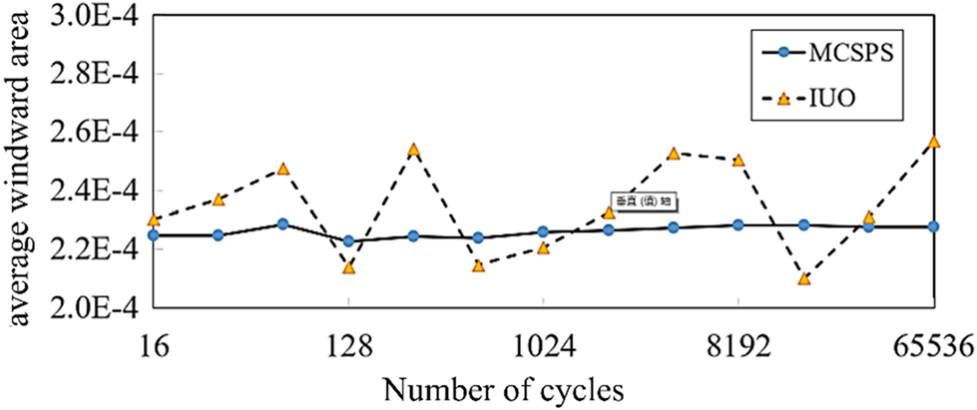
Convergence line graph of average windward areas of the MCSPS algorithm and IUO method.

The initial attitude of fragment is randomly generated. With the IUO method, 16 specific directions at the initial attitude are taken as rotation angle vectors and recorded as a cycle. With the MCSPS algorithm, 16 random directions at the initial attitude are taken as rotation angle variables and recorded as a cycle. The convergent line graph of average windward area obtained by two methods is shown in Fig. 12. As the number of cycles increases, the average windward area obtained by MCSPS algorithm gradually converges, while the windward area obtained by IUO method cannot converge. It shows that the accuracy of IUO method is greatly affected by the initial projection plane. Different initial projection planes will lead to great differences in the results of average windward area, and the calculation accuracy is not as good as that of MCSPS method.

**Figure 13:**
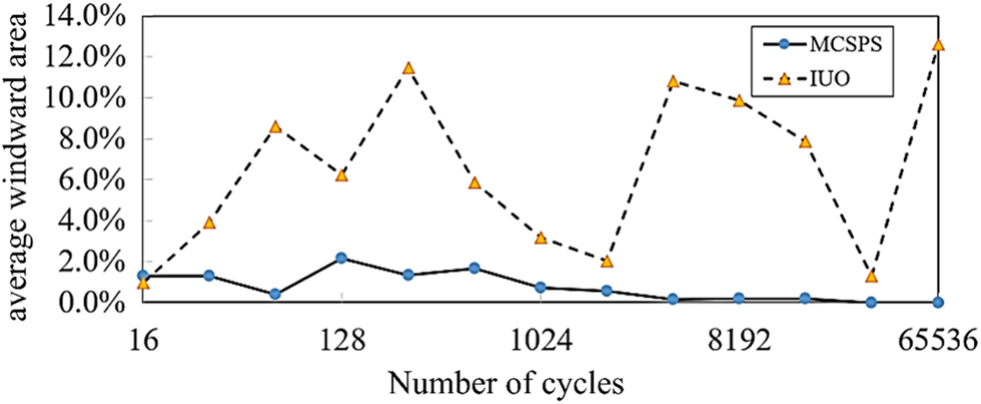
Convergence line graph of relative error of the MCSPS algorithm and IUO method.

Taking the mean value of 2^16^ (65,536) cycles of two algorithms as the approximate true value of the average windward area, the line graph of the relative error of two algorithms is shown in Fig. 13 with the increase of the number of cycles. Under the same number of cycles, the relative error of MCSPS algorithm is lower than that of IUO algorithm. The relative error of IUO algorithm does not decrease with the increase of cycle times, and its maximum relative error is 12.6%. But the relative error of MCSPS algorithm decreases gradually with the increase of cycle times, and the maximum relative error is only 2.2%.

#### Comparison of solution time

**Figure 14:**
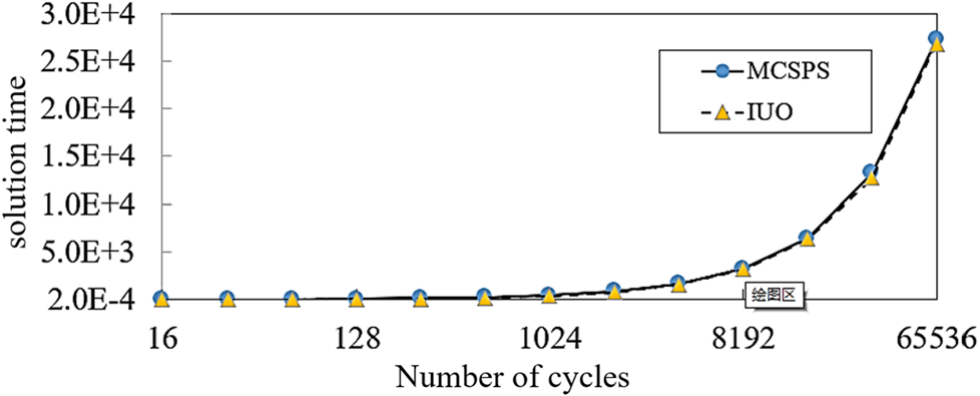
Solution time of the MCSPS algorithm and IUO method for frag 4.

The solution time of the MCSPS algorithm and IUO method are as shown in Fig. 14. The figure curve is presented on a logarithmic scale with a base of 2. With the increase of the number of cycles, the time complexities of the two algorithms are basically the same. The IUO algorithm usually needs only 16 cycles to obtain a low-precision average windward area result, whereas the MCSPS method needs thousands of cycles to obtain a high-precision result. Thus, the MCSPS algorithm has a longer calculation time than the IUO algorithm.

The comparison shows that the IUO algorithm only needs 16 cycles, and the solution time is shorter. However, due to its results are not convergent, the calculation accuracy is not high in solving irregular fragments. The MCSPS algorithm needs thousands of cycles to get a result, but the calculation accuracy is higher. For MCSPS algorithm, within the allowable calculation error range, the calculation efficiency of the algorithm can be improved by reducing the number of cycles.

#### Application comparison of equivalent modeling method and MCSPS method

**figure 15:**
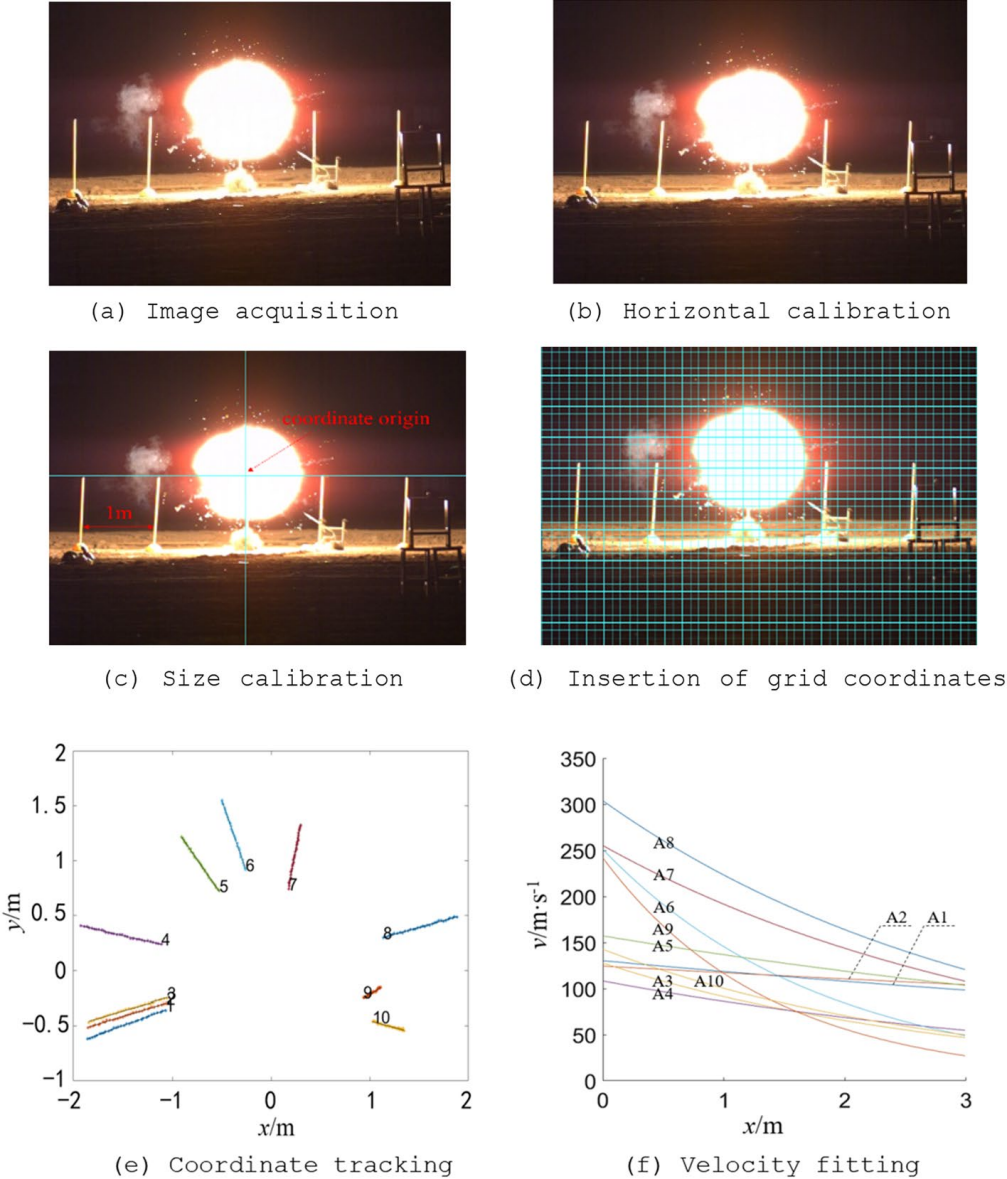
the process diagram of high speed photography method.

**Figure 16:**
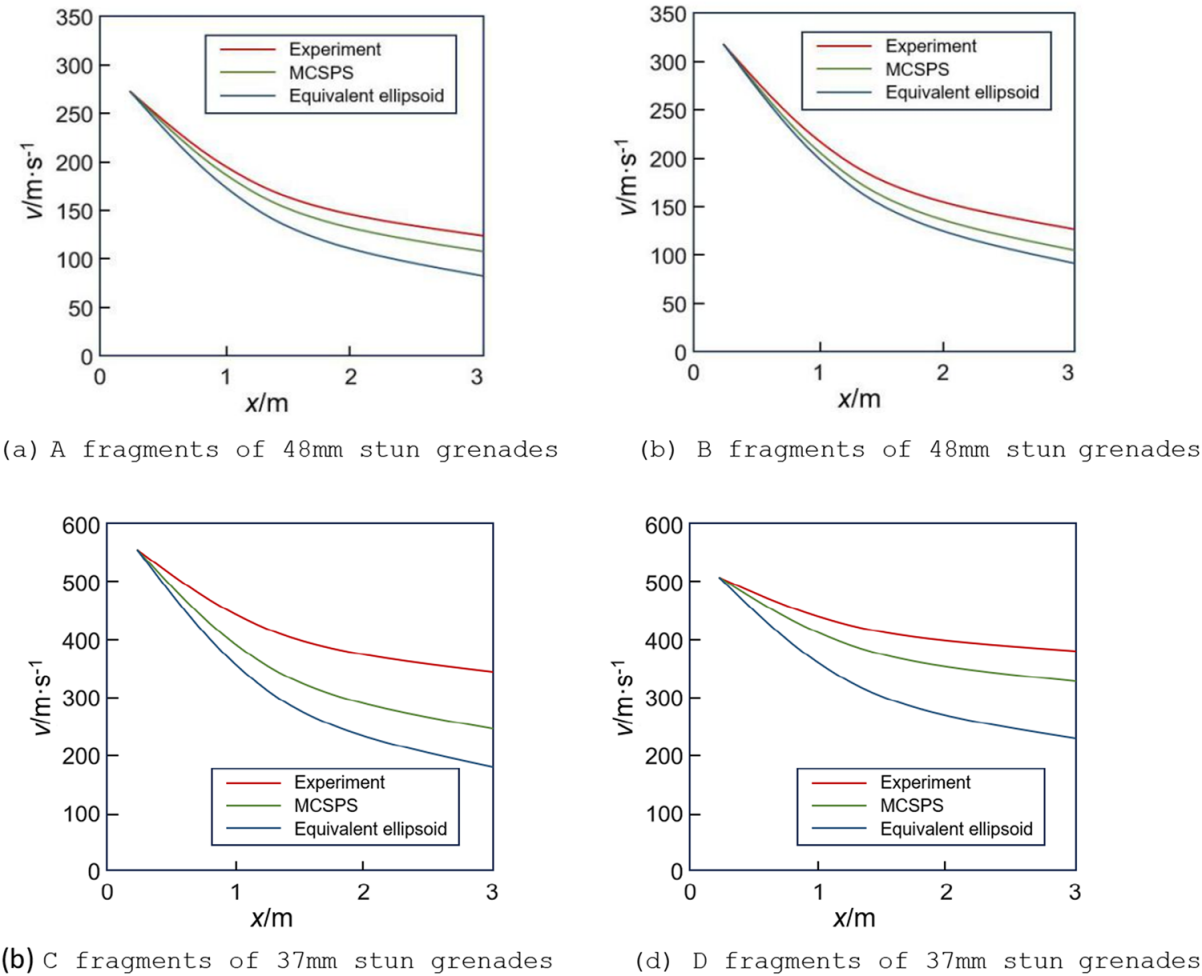
Results comparison of different methods for four stun grenades.

To verify the superiority of the MCSPS method in calculating the velocity attenuation of explosive natural fragments, the results of MCSPS method and equivalent modeling method^12^ were compared with experimental results by static explosion experiments. In the experiment, four stun grenades were used as test samples with a height of 1 m from the ground. The trajectory of fragments was recorded with high-speed photography method of 20,000 frames. After the original image underwent horizontal calibration, size calibration, insertion of grid coordinates, coordinate tracking and velocity fitting, fragment velocity attenuation curve can be obtained, and the process diagram is shown in Fig. 15. With calculating the system of particle external ballistic equations^19^ and averaging fragments velocities, average velocity attenuation curve of fragments are obtained at a distance of 1–3 m. This experimental data of four stun grenades were compared with the results obtained by MCSPS method and equivalent modeling method, as shown in Fig. 16.

In Fig. 16, it can be seen that fragment velocity attenuation curves of the MCSPS method are closer to experimental curve. The maximum velocity errors of four stun grenades at 3 m are respectively 11%, 14%, 12%, and 13% for MCSPS, while the errors of equivalent modeling method are respectively 24%, 26%, 22%, and 42%. The main reason for the results are that ellipsoidal equivalent modeling method will make equivalent windward area of each fragment larger than actual windward area, resulting in faster attenuation of fragment velocity and closer dispersion distance.

There are two main reasons for the error between MCSPS method and experimental results. Firstly, there are natural fragments with small rotating angle velocity, which makes Monte Carlo principle unable to fully reflect flipping law of such fragments; Secondly, MCSPS method uses particle external ballistic equation to calculate dispersion characteristics of fragments, which cannot characterize the impact of near-field shock waves on fragment velocity, resulting in errors between experimental test results and theoretical method results.

## Conclusion

The projection area obtained by subdivision projection algorithm is basically the same as that obtained by CAD method, with the maximum calculation error being ≤ 7%. Moreover, the algorithm accuracy is much higher than the 36% calculation error of the equivalent ellipsoid method. The results of the MCSPS algorithm are consistent with the average windward area results obtained from theoretical formula of prefabricated fragments, and the calculation error is ≤ 3%. In comparison with the IUO method, the MCSPS algorithm has better convergence and higher calculation accuracy. The MCSPS algorithm also has good generality, which provides a new method for calculating the dispersion characteristics parameters and terminal effectiveness of numerical simulation fragments automatically.

## Data Availability

The fragments model data that support the findings of this study are available from the corresponding author, [Liu Xingyu], upon reasonable request. The source code is temporarily unavailable due to personal privacy and confidentiality restrictions.
